# *Poria cocos* polysaccharides exert prebiotic function to attenuate the adverse effects and improve the therapeutic outcome of 5-FU in Apc^*Min/*+^ mice

**DOI:** 10.1186/s13020-022-00667-8

**Published:** 2022-10-03

**Authors:** Lin Yin, Guoxin Huang, Imran Khan, Lu Su, Wenrui Xia, Betty Yuen Kwan Law, Vincent Kam Wai Wong, Qiang Wu, Jingyi Wang, Wai Kit Leong, W. L. Wendy Hsiao

**Affiliations:** 1 State Key Laboratory of Quality Research in Chinese Medicine, Macau University of Science and Technology, Macao, China; 2 Clinical Research Center, Shantou Central Hospital, Shantou, China; 3 Zhuhai MUST Science and Technology Research Institute, Zhuhai, China; 4 Affiliated Foshan Maternity and Child Healthcare Hospital, Southern Medical University, Foshan, Guangdong China

**Keywords:** *Poria cocos* polysaccharides, 5-fluorouracil, Colonic cancer, Gut microbiota

## Abstract

**Background:**

As a first-line chemotherapeutic agent, 5-fluorouracil (5-FU) exhibits many side effects, weakening its efficacy in cancer treatment. In this study, we hypothesize that *Poria cocos* polysaccharides (PCP), a traditional Chinese herbal medicine with various bioactivities and prebiotic effects, might improve the therapeutic effect of 5-FU by restoring the homeostasis of the gut microenvironment and the commensal gut microflora.

**Methods:**

Apc^*Min/*+^ mice were employed to evaluate the anti-cancer effect of 5-FU in conjunction with PCP treatment. Body weight and food consumption were monitored weekly. Polyp count was used to assess the anti-cancer effect of PCP and 5-FU. Expressions of mucosal cytokines and gut epithelial junction molecules were measured using qRT-PCR. 16S rRNA gene sequencing of fecal DNAs was used to evaluate the compositional changes of gut microbiota (GM). Transplantation of *Lactobacillus johnsonii* and *Bifidobacterium animalis* were performed to verify the prebiotic effects of PCP in improving the efficacy of 5-FU.

**Results:**

The results showed that PCP treatment alleviated the weight loss caused by 5-FU treatment and reduced the polyp burden in Apc^*Min/*+^ mice. Additionally, PCP treatment eased the cytotoxic effects of 5-FU by reducing the expressions of pro-inflammatory cytokines, increasing the anti-inflammatory cytokines; and significantly improving the gut barriers by enhancing the tight junction proteins and associated adhesion molecules. Furthermore, 16S rRNA gene sequencing data showed that PCP alone or with 5-FU could stimulate the growth of probiotic bacteria (*Bacteroides acidifaciens*, *Bacteroides intestinihominis*, *Butyricicoccus pullicaecorum*, and the genera *Lactobacillus*, *Bifidobacterium*, *Eubacterium*). At the same time, it inhibited the growth of potential pathogens (e.g., *Alistipes finegoldii*, *Alistipes massiliensis*, *Alistipes putredinis*., *Citrobacter* spp., *Desulfovibrio* spp., and *Desulfovibrio desulfuricans*). Moreover, the results showed that transplantation of *L.johnsonii* and *B.animalis* effectively reduced the polyp burden in Apc^*Min/*+^ mice being treated with 5-FU.

**Conclusion:**

Our study showed that PCP could effectively improve the anti-cancer effect of 5-FU by attenuating its side effects, modulating intestinal inflammation, improving the gut epithelial barrier, and modulating the gut microbiota of Apc^Min/+^ mice.

**Supplementary Information:**

The online version contains supplementary material available at 10.1186/s13020-022-00667-8.

## Introduction

Colorectal cancer (CRC) is a high-risk illness that threatens countless people's health and causes a severe financial burden to society. With more than 1.9 million new cases and 935,000 deaths in 2020, CRC will become the second most common cause of cancer-related mortality worldwide [1]. For CRC therapy, surgery is the first-choice therapy. However, because of the high incidence of relapse and metastasis, chemotherapy still plays a vital role in caring for CRC patients in clinics. For the past 50 years, 5-FU, the derivative of uracil, has served as the classic and standard agent for CRC chemotherapy. 5-FU exerts antitumor effects by interfering with thymidylate synthase and inhibiting DNA and RNA synthesis. As a consequence, 5-FU effectively induces cell cycle arrest and apoptosis of the tumor cells [2]. However, the adverse effects of 5-FU, including gastrointestinal toxicities, peripheral blood cytopenia, and neurologic abnormalities, cannot be ignored [3]. Furthermore, among various side effects of 5-FU therapy, about 50–80% of the patients suffered severe intestinal mucosal damage that caused severe diarrhea, nausea, vomiting, and anorexia [4, 5]. Therefore, it is necessary to develop new strategies to minimize or prevent the adverse effects and improve the therapeutic efficiency of 5-FU.

The host-microbes interaction plays a crucial role in health and disease [6, 7]. Evidence pointed out that illness and drug treatment can lead to the compositional shift of GM. On the other hand, alteration of specific GM can significantly affect the host’s drug response and disease development [8]. For example, studies showed that disruption of the microbiota reduced the efficacy of tumor-bearing mice to CpG-oligonucleotide immunotherapy and platinum chemotherapy [9, 10]. Gut microbes facilitating cancer treatment also extended to the targeted immunotherapies, such as cytotoxic T-lymphocyte-associated protein 4 (CTLA-4) and anti-programmed cell death ligand 1 (anti-PD-L1) therapies [11, 12]. The influence of GM is also well refracting in CRC development and its response to drug treatment [13, 14]. For example, recent reports showed that cancer chemotherapeutic agents, including 5-FU, could increase the relative abundance of specific pathogens, e.g., *Escherichia* and *Bacteroides fragilis* [13, 15]. Another study indicated that altered gut microbiota might cause intestinal mucositis associated with 5-FU therapy [16]. Furthermore, recent reports also pointed out that dysbiosis reduces the antitumor efficacy of 5-FU [17, 18]. On the other hand, the oral administration of probiotics ameliorated 5-FU-induced mucositis in mice [19]. The study further illustrated that combined 5-FU and probiotic treatment suppressed the inflammatory cytokines triggered by the 5-FU treatment in the colon of mice [19]. Based on these results, it is a feasible strategy to improve 5-FU therapeutic effect and make safer treatment through the modulation of GM composition.

Chinese herbal medicines have been served clinically as adjuvant therapy to reduce adverse effects and improve the efficacy of chemotherapeutic agents [20, 21]. Our previous studies found certain Chinese herbal medicines, including saponins from *Ginseng, Notoginseng*, and *Gynostemma pentaphyllum;* polysaccharides from *Lycium barbarum, Ganoderma lucidum* and *Poria cocos*, exhibit prebiotic effects in both normal and diseased mouse models [22–25]. *Poria cocos* (PC, newly named *Wolfiporia cocos*) is a dietary herbal medicine commonly used to treat gastrointestinal diseases. Polysaccharide is the principal constituent and the main active ingredient of PC. Evidence showed that PC polysaccharides (PCP) possess anti-cancer, anti-inflammation, anti-aging, immunomodulation, and lipid regulation properties [26–29]. Furthermore, our previous report suggests that PCP can effectively modulate GM and act as a prebiotic agent in mice [23]. In line with our study, a recent study also found that main metabolites of PC significantly altered the gut microbiota and the intestinal metabolites in mice [30]. Besides modulating the dysbiosis in the alcoholic hepatic steatosis mouse model, PCP also alleviated liver symptoms by inhibiting the ethanol-induced fungal overgrowth [31]. Here, we hypothesize that PCP might exert its prebiotic effects to minimize the adverse effects and improve the anti-cancer effect of 5-FU.

In this study, the colonic cancer model Apc^*Min/*+^ mice were treated with 5-FU with and without PCP. Polyps counting was used to evaluate the anti-cancer effect of 5-FU with or without PCP. In addition, 16S rRNA gene sequencing of fecal DNAs, inflammatory cytokines test, immunohistochemistry, and fecal microbial transplantation (FMT) were conducted to investigate the potential prebiotic effects of PCP in alleviating adverse effects of 5-FU by modulating the GM and gut microenvironment of Apc^*Min/*+^ mice.

## Materials and methods

### Animals and treatments

**Fig. 1:**
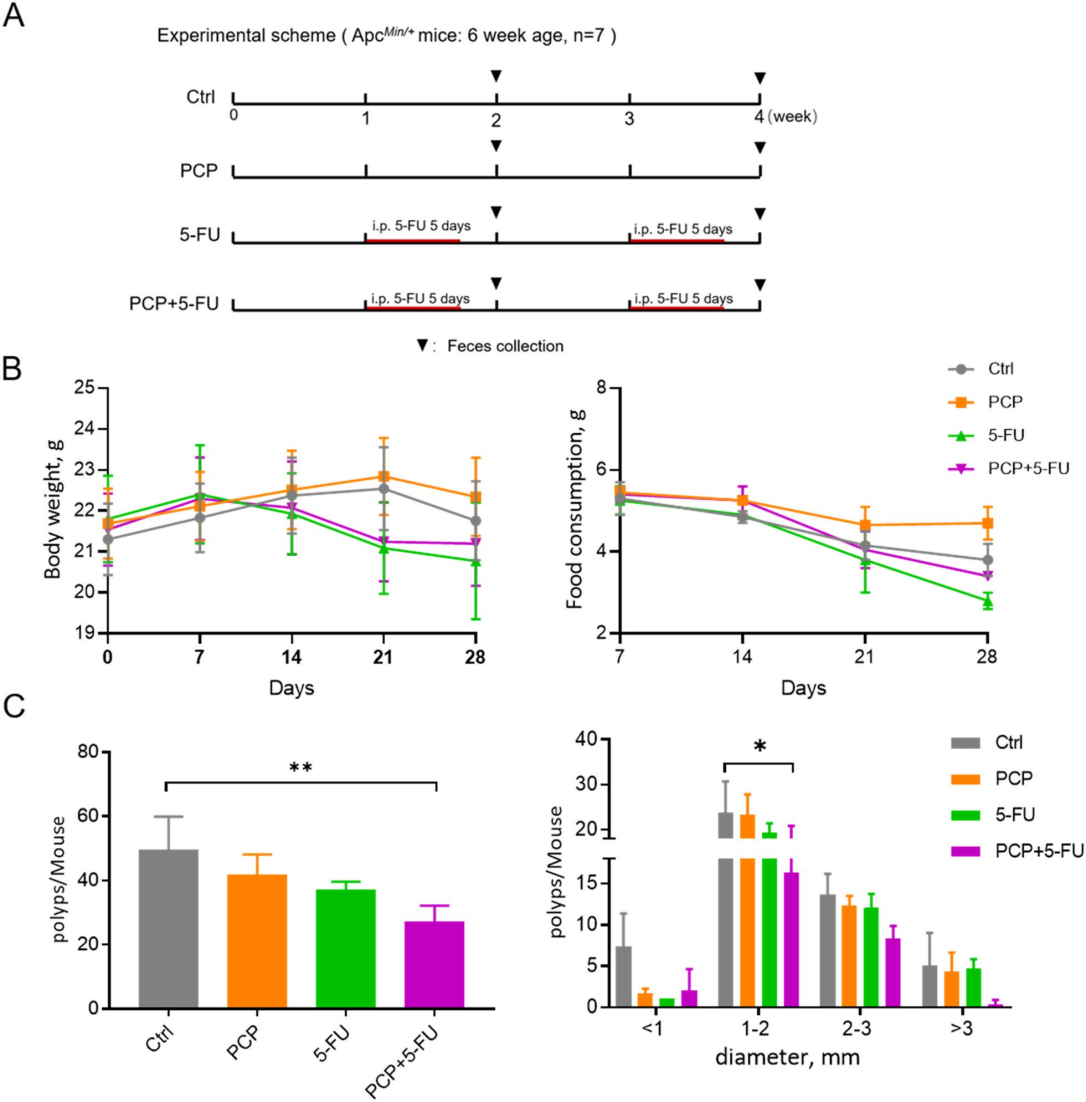
PCP enhanced the anti-cancer effects of 5-FU in Apc^*Min/*+^ mice. **A** The treatment scheme; **B** The experimental mice’s body weight and food consumption. **C** Effect of PCP and 5-FU on the total number of polyps; and the number and size distribution of the intestinal polyps. The data are presented as the mean ± SD. *p < 0.05, ** p < 0.01, n = 7

The Apc^*Min/*+^ mice (age 6–8 weeks) were purchased from Jackson's Laboratory and bred in-house for heterozygous mice. The genotype of Apc^*Min/*+^ mice was identified using KAPA Mouse Genotyping Kit (Roche, USA). Mice were fed with PicoLab^®^ Rodent Diet 20-5035 (LabDiet, USA). Mice were housed in a 12-h/12-h dark–light cycle facility and kept in the IVC equipment with free access to food and water. 28 Apc^*Min/*+^ mice were randomly divided into four groups, i.e., the control group, PCP group, 5-FU group, and PCP + 5-FU group. Dry powder of PCP was dissolved in sterile distilled deionized (DD) water. The mice were gavage daily with 750 mg/kg of PCP or solvent control for four consecutive weeks. In addition, mice in the 5-FU and PCP + 5-FU groups were injected intraperitoneally with 40 mg/kg of 5-FU for five successive days in the 2nd and 4th weeks (Fig. 1A). Food consumption and body weight were recorded weekly. At the end of the experiment, the mice were euthanized and sacrificed following the approved guidelines of the Ethics Review Committee for Animal Research of the Macau University of Science and Technology.

### Preparation of PCP herbal extracts

Water-soluble polysaccharides of *Poria cocos* (the content of polysaccharide is ≥ 30%) were purchased from Jiangsu Goodex Mushroom Biotech Co. Ltd. (Yancheng, Jiangsu, China). The quantitation of the PCP was performed as in the previous description [23]. PCP powder was dissolved in dd-water for the later experiments. 5-FU was purchased from Shanghai Macklin Biochemical Co., Ltd. (Shanghai, China).

### Fecal samples collection and extraction of genomic DNA

Fecal samples were collected in the 2nd and 4th weeks of the experiment and stored at −80 ℃ for the later experiments. The total DNA was extracted using QIAamp DNA Stool Mini Kit (QIAGEN, German) based on the manufacturer's protocol.

### Animal dissection and polyp counting

The mice were terminated and dissected at the end of the experiment for polyp counting. Section of intestine from the cecal junction was collected, rinsed with cold PBS, and fixed in 10% formalin for the tissue section. Mucosal samples were collected from the colon and distal small intestine and frozen for later biochemical analyses. The rest portion of the intestine was cut open longitudinally, rinsed with cold PBS, and fixed in 10% formalin for 48 h, then stained with methylene blue. The number and size of the polyps were scored under the Olympus SZX10 microscope.

### Total RNA preparation and quantitative reverse transcription polymerase chain reaction (qRT-PCR).

The total RNAs were extracted from mucosal samples using TRNeasy Mini Kit (QIAGEN, Hilden, German), following the procedures from the manufacturer. The concentration of total RNA was determined by Nanodrop 2000C spectrophotometer (Thermo, USA). Following our previous description, the qRT-PCR reaction was carried out using Applied Biosystems ViiATM 7 PCR system (Carlsbad, CA, USA) [32]. β-actin was used as the internal control to normalize the PCR reaction of each specific marker. The 2^−ΔΔCt^ method was applied to calculate the fold change of relative gene expression. ΔΔCt = (Ct_treatment_target gene_—Ct_treatment_reference gene_)–(Ct_control_target gene_—Ct_control_reference gene_). qRT-PCR was used to measure the expression of mucosal pro-inflammatory cytokines (IL-1β, IL-6, IL-18, INOS, TNF-α, and FOXP3) and anti-inflammatory cytokines (IL-4, IL-10, IL-12, and IL-13); tight junction proteins (ZO-1 and occluding) and adhesion molecules (VCAM-1, ICAM-1, E-cadherin, and N-cadherin). Specific primer sets were applied to quantify *B. animalis* and *L. johnsonii* using qPCR as described [32]. The primer sets used for the PCR analysis were listed in the Additional file 1: Table S1.

### Histology and immunohistochemistry staining

H&E, Alcian blue staining, and immunohistochemical (IHC) staining were performed with 5 µm thick paraffin sections following the standard protocol. Specific antibodies for IHC staining included the anti-lysozyme antibody (1:200, A0099, DAKO), E-cadherin (1:200, #3195S, CST), N-cadherin (1:100, 13,116, CST), ZO-1 (1:200, 61–7300, Invitrogen), occludin (1:200, 40–4700, Invitrogen). VECTASTAIN^®^ Elite^®^ ABC Universal Kit (PK-6200, Vector) was used to hybridize the mentioned antibodies. Tissue sections were mounted and viewed under the Leica microscope. The images were taken with the Leica camera (DFC310 FX) and the Leica Application Suite software (Version 4.4.0, Switzerland).

### Fecal DNA preparation, 16S RNA gene sequencing, and data analysis

For fecal GM analysis, sequencing of total genomic DNA was carried out using Illumina MiSeq (Illumina, San Diego), targeting the V3–V4 region of the 16S rRNA genes with barcoded 515F and 806R universal primers [33]. The detailed sequencing procedures were performed as previously described [34].

### In vitro culture and transplantation of *Lactobacillus johnsonii* and *Bifidobacterium animalis*

*L. johnsonii* (1.3348, China General Microbiological Culture Collection Center) and *B. animalis* (1.1259, Guangdong Microbial Culture Collection Center) were acquired and cultured in the designated growth medium (Additional file 1: Table S2) and kept in an anaerobic chamber (Whitley A35 Workstation, Don Whitley Scientific Limited, UK) in 5% CO_2_, 10% H_2_, and 85% N_2_ according to *Liao *et al*., 2021* [35]. Bacteria were collected by centrifugation at 5000 rpm for 5 min and diluted to 1 × 10^9^ cells/ml for further experiments. In the microbes transplantation experiment, 20 Apc^*Min/*+^ mice (6–8 weeks old) were randomly divided into four groups: vehicle control, 5-FU, *B. animalis* + 5-FU, and *L. johnsonii* + 5-FU. 2 × 10^8^ live bacteria from each species *were* gavaged to mice every other day for four consecutive weeks (Fig. 6B). 5-FU at 40 mg/kg was injected intraperitoneally to mice for five successive days in the 2nd and 4th weeks.

### Statistical analysis

The statistical analysis and graphical presentation of data were performed by GraphPad Prism (8.0). The significant changes were determined by One-way ANOVA (for parametric data) and Kruskal–Wallis (non-parametric data) tests. Bonferroni and Dunn-Bonferroni tests were performed for parametric and non-parametric multiple comparison p values correction.

## Results

### PCP treatment enhanced the anti-cancer effects of 5-FU in Apc^*Min/*+^ mice

To investigate the potential synergistic effect of PCP toward the anti-cancer effect of 5-FU, 6–8 weeks old Apc^*Min/*+^ mice were treated with 5-FU with or without PCP for four weeks. The treatment scheme is illustrated in Fig. 1A. There were no significant differences in body weight and food consumption among the groups in the 1st and 2nd weeks. However, the body weight and food consumption of the 5-FU and PCP + 5FU groups started to decline from the 3^rd^ week and on. The decline was minimized in the group co-administrated PCP and 5-FU by the end of the experiment (Fig. 1B). Importantly, PCP treatment enhanced the anti-cancer effect of 5-FU based on the total number and the size distribution of the intestinal polyps in the mice (Fig. 1C).

### PCP improved the intestinal epithelial barrier of the control and 5-FU-treated Apc^*Min/*+^ mice

**Fig. 2:**
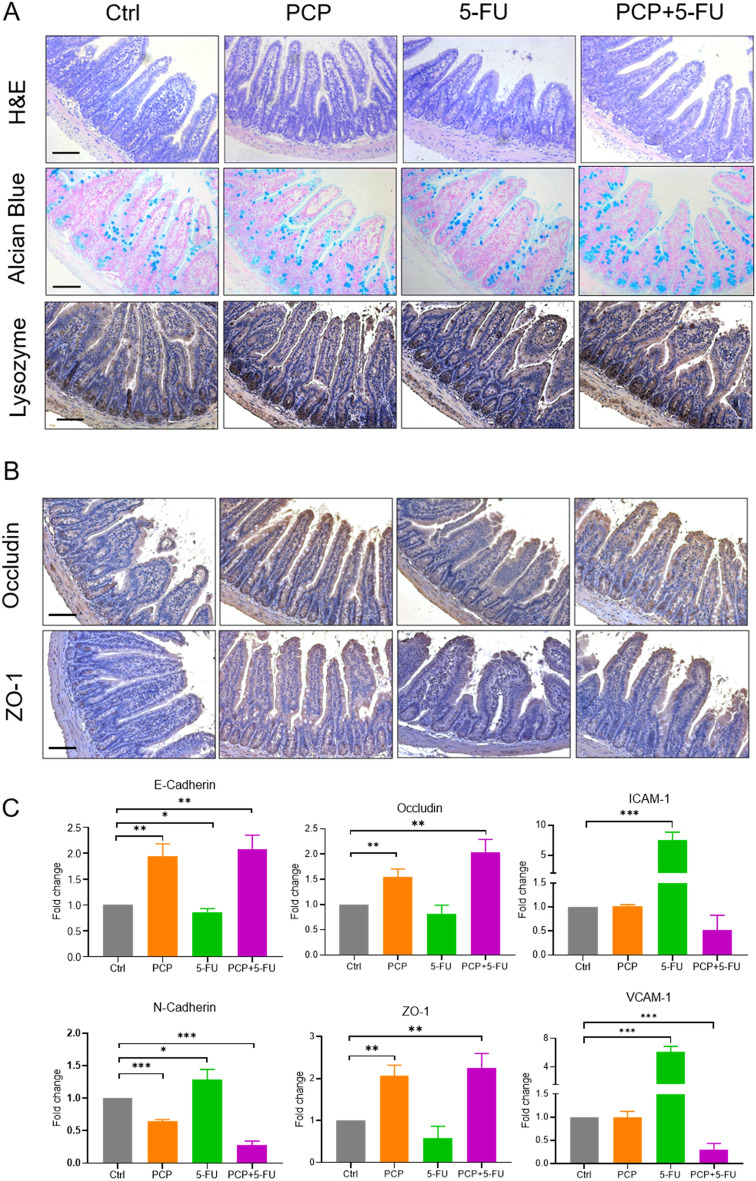
PCP improved the intestinal epithelial barrier damaged by 5-FU. **A** H&E, Alcian blue, and lysozyme stains of the intestine. Alcian blue staining was used to detect the goblet cells. Anti-lysozyme staining was used to detect Paneth cells. The dark brown staining at the bottom of the crypts indicates the location of Paneth cells; **B** IHC staining of occluding and ZO-1. The positive stains were presented in dark brown staining. Scale bar = 100 μm; **C** The mRNA expressions of E-cadherin, N-cadherin, occluding, ZO-1, ICAM-1, and VCAM-1 by qRT-PCR; Data are presented as the mean ± SD, n = 3. *p < 0.05, ** p < 0.01, *** p < 0.001

The cancer-prone Apc^*Min/*+^ mice are known to carry dysfunction of the gut barrier and appear to be associated with spontaneous intestinal polyps formation [36]. The H&E staining of the intestinal villi showed various degrees of damage in the Ctrl and 5-FU groups compared to the PCP and PCP + 5-FU groups (Fig. 2A). Moreover, Alcian blue and lysozyme staining revealed that the number of Paneth cells and goblet cells decreased in the Ctrl and 5-FU groups. PCP alone or co-treatment with 5-FU restored the number of Paneth cells and goblet cells (Fig. 2A, Fig. S1). IHC staining and qRT-PCR showed that tight junction molecules occludin and ZO-1 were at low levels in the Ctrl and 5-FU groups and markedly elevated upon PCP treatment in the PCP and PCP + 5-FU mice (Fig. 2B and )C). In addition, the mRNA expressions of ICAM-1 and VCAM-1 were significantly induced upon 5-FU treatment, but treatment with PCP + 5-FU resumed the level close to the untreated control (Fig. 2C). We also evaluated the expression of the adhesion molecules, including E-cadherin and N-cadherin. Their expressions are the makers of cancer progression and prognosis. The results showed that 5-FU treatment decreased E-cadherin but increased N-cadherin. On the other hand, either PCP or PCP-5-FU treatment substantially enhanced the expressions of E-cadherin and downregulated N-cadherin in the treated mice (Fig. 2C).

### PCP reversed the inflammatory gut environment disturbed by 5-FU treatment

**Fig. 3:**
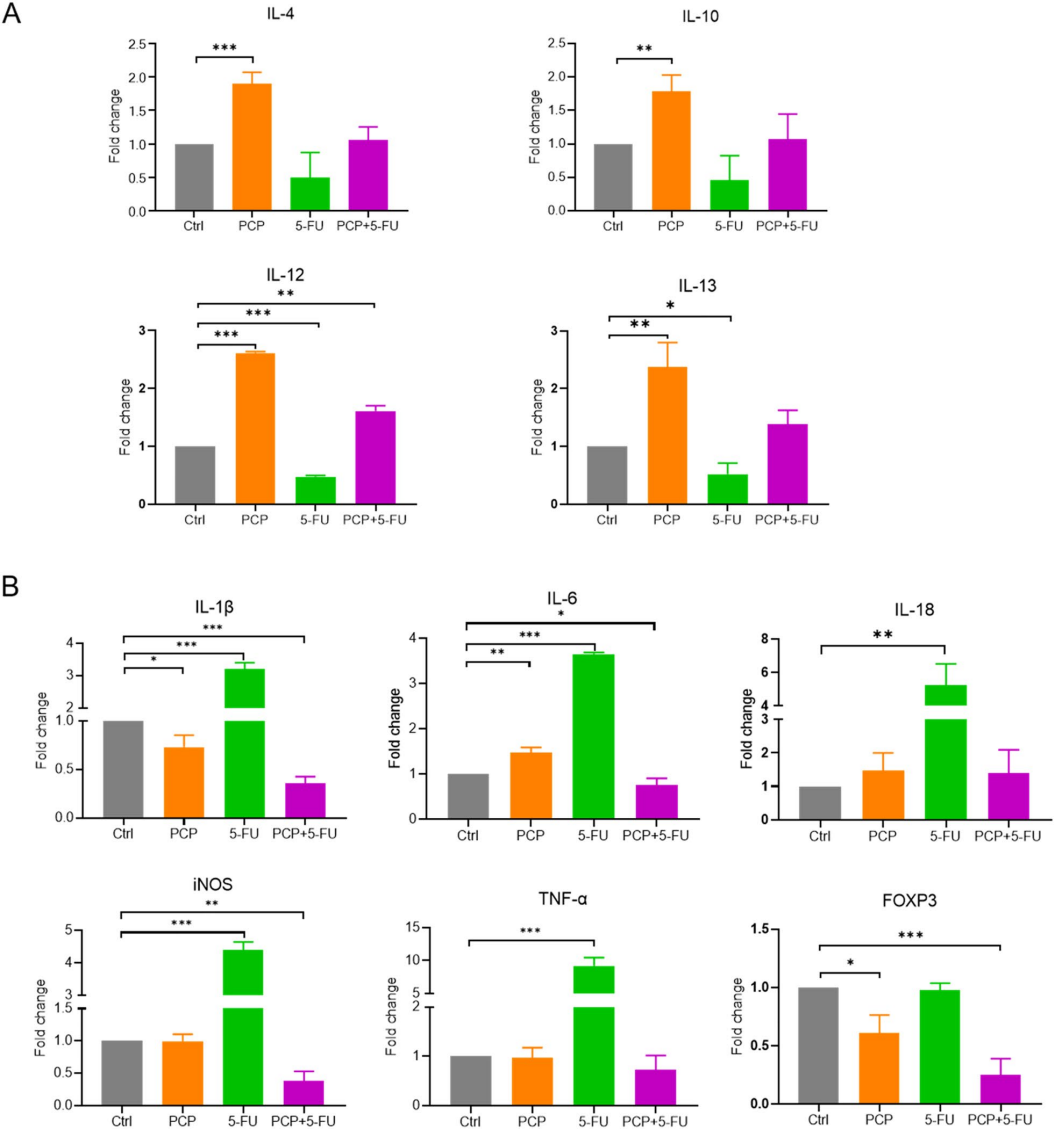
Expressions of mucosal cytokines in the guts of the mice treated with PCP, 5-FU, or PCP + 5-FU. The expressions of anti-inflammatory cytokines (**A**) and pro-inflammatory cytokines (**B**) were measured by qRT-PCR. The data are presented as the mean ± SD, n = 3. *p < 0.05, ** p < 0.01, *** p < 0.001

Stimulation of intestinal inflammation and causing mucositis are the main side-effects of 5-FU. Higher expression of pro-inflammatory cytokines is usually involved in the progression of mucositis. Therefore, we evaluated the expressions of the mucosal pro-inflammatory and anti-inflammatory cytokines using the qRT-PCR test. Our results showed that treatment with PCP alone markedly (p < 0.01) increased the expression of anti-inflammatory cytokines (IL-4, IL-10, IL-12, and IL-13) compared to the control mice (Fig. 3A). Conversely, 5-FU treatment decreased anti-inflammatory cytokines' expression while drastically increasing pro-inflammatory cytokines' expression (Fig. 3A and B). Results also clearly showed that the intervention of PCP can overturn the upregulated IL-1β, IL-6, IL-18, iNOS, TNF-α, and FOXP3, and the down-regulated IL-4, IL-10, IL-12, and IL-13 that caused by the treatment of 5-FU (Fig. 3A and B).

### PCP alone and in combination with 5-FU modulated GM diversity and composition of the mice

**Fig. 4:**
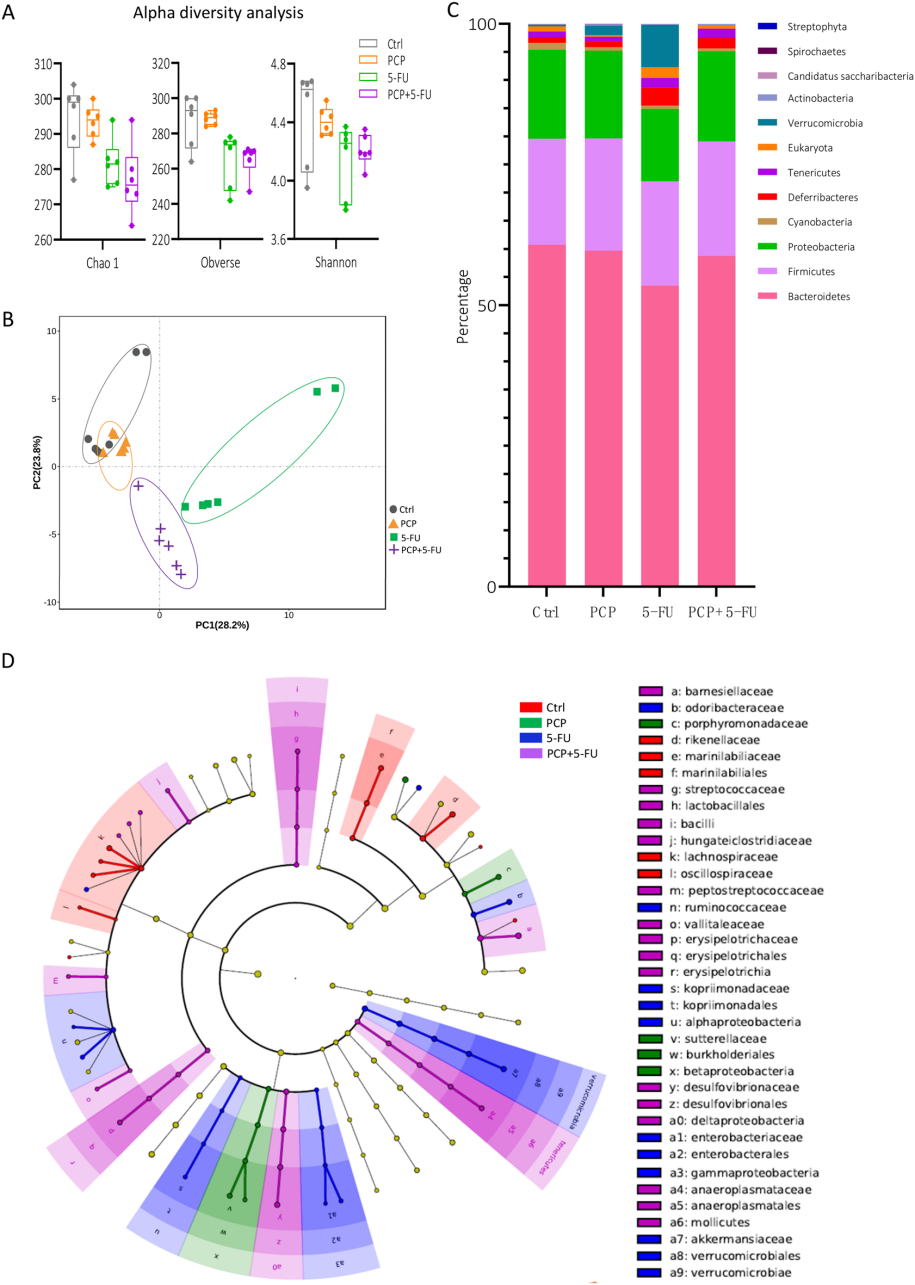
PCP treatment modulated the gut microbial diversity and composition in mice treated with 5-FU. **A** Alpha diversity analysis of the gut microbiota from each group. **B** PCA plots are applied to display the clustering of gut microbiota. **C** Average relative abundance of the dominant phyla. The y-axis represents the average percentages of OTUs reads. Different colors display significant changes in abundant phylum taxa of different groups. **D** The overall exhibition of LEfSe analysis by using taxonomic cladograms

To investigate the role of PCP in regulating GM diversity and composition of mice treated with 5-FU, fecal genomic DNA was extracted from the mice and executed 16S rRNA gene sequencing. Alpha-diversity analysis showed that mice treated with 5-FU alone or combined with PCP lowered the diversity and richness of GM compared to the control and PCP groups (Fig. 4A). Moreover, the PCA plot showed the 5-FU and 5-FU + PCP groups deviated from the Ctrl and PCP clusters. However, PCP intervention brought the 5-FU cluster closer to the control group (Fig. 4B). The taxonomic comparison showed that *Bacteriodetes*, *Firmicutes*, and *Proteobacteria* were the dominant phyla among all the experimental groups (Fig. 4C). 5-FU treatment decreased the relative abundance of *Bacteroidetes*, while clearly increasing the relative abundance of *Deferribacteres*, *Eukaryota*, and *Verrucomicrobia* (Fig. 4C). LEfSe analysis showed that the increase of the *Verrucomicrobia* contributed to the enrichment of the family of *Akkermansiaceae* (Fig. 4D). Besides, the families of *Enterobacteriaceae* and *Gammaproteobacteria* were significantly increased in the 5-FU group (Fig. 4D). As a note, expansion of the *Enterobacteriaceae* is associated with inflammation and CRC [37]. Additionally, the family of *Lactobacillales* was significantly enriched in the PCP + 5-FU group, which could result from the enrichment of *Lactobacillus* (Fig. 4D). We also observed enhancement of the families *Barnesiellaceae* and *Anaeroplasmataceae* in the PCP + 5-FU group (Fig. 4D).

### PCP alone or co-administration with 5-FU increased the relative abundance of beneficial bacteria while decreased the potential pathogens

**Fig. 5:**
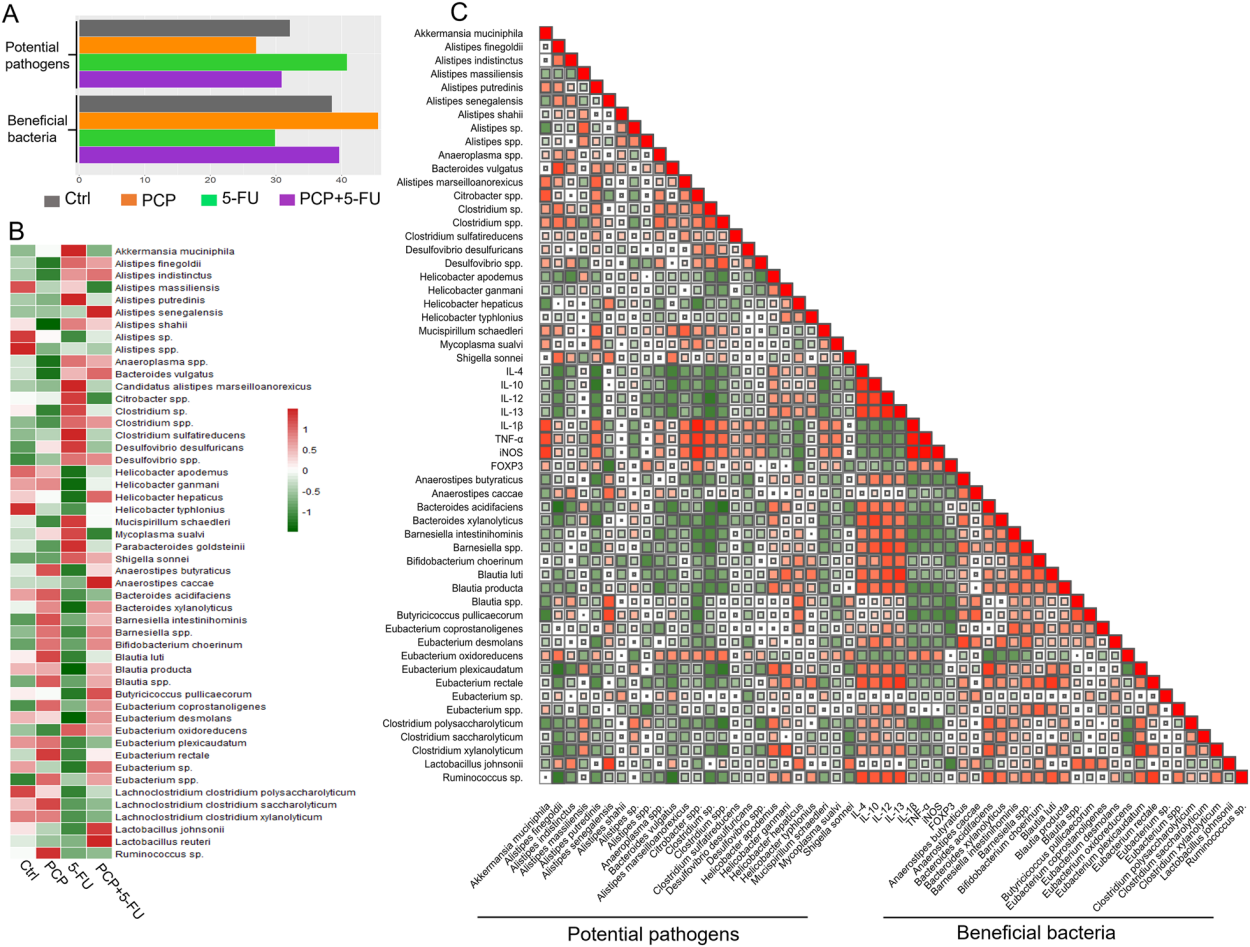
The effects of PCP treatment on beneficial and potential pathogens and the correlation between bacteria and the expression of inflammatory cytokines. **A** The proportion of potential pathogens and beneficial bacteria from detected bacteria. **B** Heatmap showing the relative abundance in the respective groups. **C** Pearson’s correlation among the bacterial species and inflammation-associated cytokines

According to the previous reports, 5-FU tends to increase the relative abundance of potential pathogens while inhibiting beneficial bacteria growth. Our result showed that PCP treatment alone or with 5-FU boosted the total beneficial bacteria while suppressing the potentially pathogenic bacteria in the treated mice (Fig. 5A, Additional file 1: Tables S3, and S4). Heatmap analysis showed that 5-FU stimulated the growth of certain potential pathogenic bacteria, including *Alistipes finegoldii*, *Alistipes massiliensis*, *Alistipes spp*., *Citrobacter* spp., *Desulfovibrio* spp., and *Desulfovibrio desulfuricans*. PCP intervention, by and large, reversed the effect of 5-FU on these potential pathogens (Fig. 5A and B). Additionally, some beneficial bacteria, such as *Bacteroides acidifaciens*, *Bacteroides intestinihominis*, *Bifidobactrium choerinum, Butyricicoccus pullicaecorum*, *Lactobacillus johnsoni*, *Eubacterium* spp. were reduced in the 5-FU treated mice; while increased in the PCP and PCP + 5-FU groups (Fig. 5B). Moreover, Pearson's correlation analysis revealed a clear positive correlation between the potential pathogens and the pro-inflammatory cytokines (IL-1β, TNF-α, iNOS, and FOXP3). And a negative correlation between the potential pathogens and the anti-inflammatory cytokines (IL-4, IL-10, and IL-12). Interestingly, the results also showed a positive relationship between the beneficial bacteria and the anti-inflammatory cytokines and a negative relationship between the beneficial bacteria and the pro-inflammatory cytokines (Fig. 5C).

### Colonization of *Bifidobacterium animalis* and *Lactobacillus johnsonii* reduced the polyp counts in Apc^*Min/*+^ mice

**Fig. 6:**
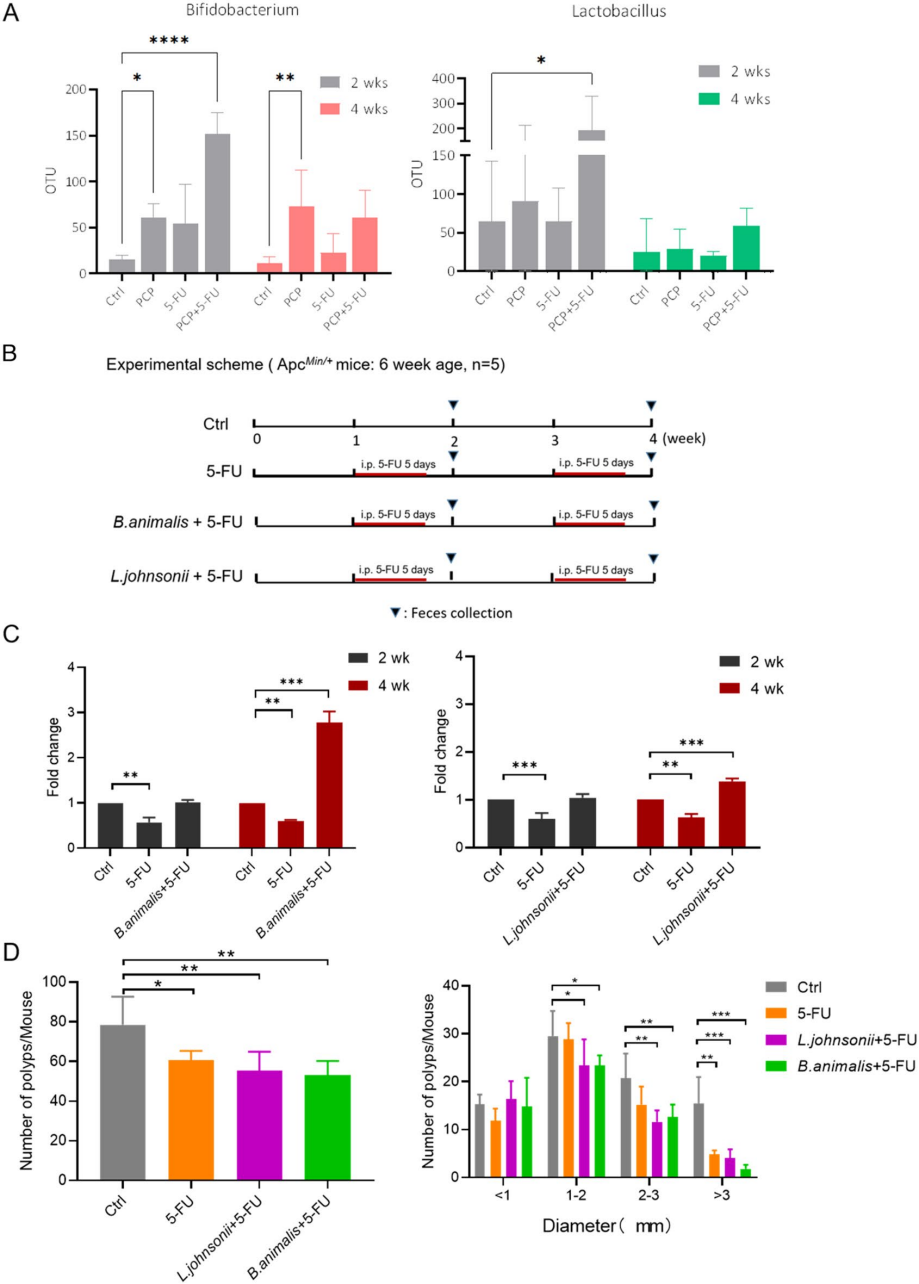
Colonization of *B. animalis* and *L. johnsonii* improved the anti-cancer effect of 5-FU in Apc^*Min/*+^ mice. **A** Presentations of the OTUs taxa of *Bifidobacterium* and *Latobacillus* in 2 and 4 weeks. **B** The treatment schemes. Detailed procedures are listed under M&M; **C** Measurement of colonized *B. animalis* (left panel) and *L. johnsonii* (right panel) in the guts of four groups of experimental mice. Quantitation of bacteria was performed by qPCR on fecal DNA collected 2 and 4 weeks from each treatment group; **D** The total number and the distribution of polyps in the experimental mice. Data are presented as the mean ± SD, n = 5. *p < 0.05, ** p < 0.01, *** p < 0.001

To further explore whether PCP-induced genera are responsible for the improvement of the anti-cancer efficacy of 5-FU, we selected the candidate bacteria for bacteria transplantation. From the 16S sequencing data, we identified two bacteria genera, *Bifidobacterium* and *Lactobacillus,* that displayed significant responses to PCP treatment, alone or in the presence of 5-FU. At the 4-week treatment time point, we observed diminishing OTUs of *Bifidobacterium* and *Lactobacillus* in most experimental groups (Fig. 6A). This phenomenon might be associated with the increased number of polyps in the mice. Based on these data, we selected two bacteria from each genus, the *Bifidobacterium animalis* and *Lactobacillus johnsonii,* to engage in the implantation experiment. The colonization of the implanted bacteria was monitored by PCR with each specific primer sequence. The data showed that both implanted bacteria successfully colonized the recipient Apc^*Min/*+^ (Fig. 6C). The data also showed that 5-FU suppressed the growth of *B. animalis* and *L. johnsonii* (Fig. 6C). Moreover, transplantation of *B. animalis* and *L. johnsonii* significantly reduced the numbers and size of the polyps compared to the untreated control and 5-FU groups (Fig. 6D).

## Discussion

5-FU is a common chemotherapeutic agent for treating various cancers, especially colon cancer. While treating cancer, 5-FU therapy also evokes multiple adverse effects in patients and hampers the efficacy of the treatment. In this study, we present evidence that the adverse effects of 5-FU can be circumvented by co-treatment with polysaccharides derived from the dietary herbal medicine *Poria cocos,* in Apc^*Min/*+^ mice. First, we found that PCP could effectively alleviate the weight loss and food consumption reduction associated with 5-FU treatment. In addition, PCP also significantly improves the efficacy of 5-FU against polyp formation in mice treated with PCP + 5-FU (Fig. 1). To understand the enhancing effect of PCP on the effectiveness of 5-FU, we examined intestinal integrity, immune response, and gut microbial composition in mice given various treatments.

The gut aligns with an epithelial layer overlaid with a mucosal layer for housing symbiotic microorganisms and suppressing the access pathobionts. The integrity of the intestinal epithelial barrier is guided by the tight junctions of the epithelial cells. The destruction of intestinal barrier integrity increases intestinal permeability, leading to microbes invasion, exacerbating inflammation, and promoting CRC risk. Our study showed that 5-FU treatment could decrease the expression of the intercellular cell adhesion protein complex and reduce the number of goblet and Paneth cells. On the other hand, PCP treatment restored the function of goblet cells and Paneth cells, enhanced the expression of intercellular cell adhesion protein complex, and further stabilized and rebuilt the intestinal epithelial barrier. Moreover, PCP effectively reversed the shift of N- to E-cadherin for the control and 5-FU treated Apc^*Min/*+^ mice. High N-cadherin and low E-cadherin indicate poor prognosis and a hallmark of epithelial-mesenchymal transition of tumors [38].

Macrophages are the most prominent innate immune cells in the gut and carry two phenotypical types. M1 macrophages produce proinflammatory cytokines such as TNF-α, IL-1β, IL-6. In contrast, M2 macrophages produce anti-inflammatory cytokines such as IL-4, IL-10, and IL-13. However, the phenotypic features of macrophages are extremely plastic and can be shifted upon microenvironmental signals. The imbalance of M1/M2 macrophages is associated with various pathological conditions [39]. Apc^*Min/*+^ mice are known to inherit inflamed intestinal tract [36]. In this study, we found that 5-FU treatment worsened the state of the intestinal tract of Apc^*Min/*+^ mice by lowering the anti-inflammatory cytokines while markedly boosting the pro-inflammatory cytokines compared to the untreated control. This finding is aligned with the frequent recurrence of intestinal mucositis in patients undergoing 5-FU [5]. Interestingly, PCP treatment reversed the shift by reducing the expression of pro-inflammatory cytokines (e.g., TNF-α, IL-1β, IL-6, and iNOS) and increasing the expression of anti-inflammatory cytokines (e.g., IL-4 IL-10, and IL-13) compared to the control group (Fig. 3). Our results echo the previous reports that PCP possesses anti-inflammatory, anti-cancer, and immunomodulation effects [26–28].

CRC is a multifactorial disease influenced by both genetic and environmental factors. However, a large body of literature has demonstrated the role of gut microbiota in cancer initiation and progression [40]. This study detected a few signature gut bacteria associated with inflammation. At the phylum level, 5-FU treatment significantly increases the relative abundance of *Verrucomicrobia*, and the increment contributed to the marked increase of *Akkermansia muciniphila* (Fig. 4C and 5B). *A. muciniphila* was enriched in CRC patients and designated as a CRC biomarker [41]. The *Deferribacteres* was also elevated in the 5-FU-treated mice in our study. We found that the increment is mainly contributed to the increase of the *Desulfovibrio desulfuricans* (Fig. 4B and 5B). *Deferribacteres*, a sulfate-reducing bacteria (SRB), has been linked to intestinal inflammation in different mouse models [32, 42, 43]. SRB produces hydrogen sulfide, a potential genotoxic, cytotoxic agent, and can cause inflammation and cancer in the gut [44, 45]. Moreover, 5-FU increased the growth of a few other potential pathogens, e.g., *Shigella sonnnei*, *Alistipes *spp., and *Citrobacter* spp., that were closely associated with gastrointestinal diseases [46–49]. Meanwhile, 5-FU treatment decreased a few SCFA-producing bacteria, such as *Bacteroides intestinihominis*, *Butyricicoccus pullicaecorum*, *Lactobacillus johnsoni*, *Bifidobactrium choerinum*, *Eubacterium *sp. SCFAs-producing bacteria plays essential roles in maintaining gut epithelial barrier integrity and intestinal immunity [50]. Our findings are in line with other reports in which 5-FU is found upregulating potential pathogens while inhibiting the beneficial bacteria in the gut [15, 51, 52]. Notably, our data showed that the intervention of PCP could reverse the disturbed GM composition. Furthermore, the combined treatment of PCP and 5-FU significantly increased the beneficial bacteria while decreasing potential pathogens compared with 5-FU alone. These results imply that one of the mechanisms of PCP to improve the anti-cancer effect of 5-FU might be through its modulation of the dysbiosis in the 5-FU treated Apc^*Min/*+^ mice. In the microbial transplant experiment, we demonstrated that colonization of *L. johnsonii* or *B. animalis* enhance the anticancer effect of 5-FU (Fig. 6D). We also observed that 5-FU treatment significantly reduced the colonization of both implanted bacteria, reiterating the overall toxic effects of 5-FU on beneficial bacteria, especially on SCFA-producing bacteria. SCFAs are the important metabolites from GM fermentation. Many studies showed that SCFAs possess prominent bioactivities in anti-inflammatory reactions, gut barrier maintenance, and renew the colonic epithelia cells [53–55]. Our previous study showed that activation of genes encoded biogenesis and metabolic functions in *B. animalis* might have contributed to the reduction of polyps burdening in Apc^*Min/*+^ mice [35]. Among many probiotic functions [56], an early report revealed that *L. johnsonii* was deficient in the cancer-prone mouse colony, while higher in abundance in a more cancer resistant mouse colony. The study further showed that administration of *L. johnsonii* reduced inflammation and genotoxicity to mice from the cancer-prone colony [57].

## Conclusions

PCP alleviates the adverse effects and improves the efficacy of 5-FU in Apc^*Min/*+^ mice by improving gut barrier integrity, reversing the inflammatory immune response, and balancing the gut microflora's dysbiosis. In addition, PCP alone exerts a strong prebiotic effect on the polyp baring Apc^*Min/*+^ mice. Our findings imply that PCP has great potential to serve as an adjuvant drug in enhancing the efficacy of 5-FU as a chemotherapy agent for CRC and other cancer types.

## Supplementary Information

**Additional file 1:** Additional data are provided in the supplementary file named Additional file 1. **Table S1.** The primer sequences of qPCR assays. **Table S2.** The formulation of the basic bacterial growth medium. **Table S3.** The relative abundance of potential pathogens. **Table S4.** The relative abundance of beneficial bacteria. **Figure S1.** The quantitation of goblet cells and Paneth cells.

## Data Availability

16S amplicon data and analysis are deposited in the CNCB (BioProject: PRJCA009723).

## Abbreviations

5-FU: 5-Fluorouracil
Apc: Adenomatous polyposis coli
PCP: *Poria cocos* Polysaccharides
CRC: Colorectal cancer
GM: Gut microbiota
FMT: Fecal microbial transplantation
IL: Interleukin
iNOS: Inducible nitric oxide synthases
TNF-α: Tumor necrosis factor-α
FOXP3: Forkhead box P3 protein
ZO-1: Zonula occludens-1
VCAM-1: Vascular cell adhesion molecule-1
ICAM-1: Intercellular adhesion molecule-1
IHC: Immunohistochemical
SRB: Sulfate-reducing bacteria
SCFA: Short chain fatty acid
