# Supplementary material for: Poria cocos polysaccharides exert prebiotic function to attenuate the adverse effects and improve the therapeutic outcome of 5-FU in ApcMin/+ mice
Source: Chin Med. 2022 Oct 3;17:116. doi: 10.1186/s13020-022-00667-8 (PMC9531437; doi:10.1186/s13020-022-00667-8)
Supplement: Supplementary file 1 — Additional file 1: Additional data are provided in the supplementary file named Additional file 1. Table S1. The primer sequences of qPCR assays. Table S2. The formulation of the basic bacterial growth medium. Table S3. The relative abundance of potential pathogens. Table S4. The relative abundance of beneficial bacteria. Figure S1. The quantitation of goblet cells and Paneth cells. [file 13020_2022_667_MOESM1_ESM.docx]

**Supplementary materials**

**The prebiotic function of *Poria cocos* polysaccharides attenuates the adverse effects and improves the therapeutic outcome of 5-FU in Apc*^Min/+^* mice**

Lin Yin^1#^, Guoxin Huang^1,2,3#^, Imran Khan^1^, Lu Su^1^, Wenrui Xia^1^, Betty Yuen Kwan Law^1^, Vincent Kam Wai Wong^1^, Qiang Wu^1^, Jingyi Wang^1^, Wai Kit Leong^1^, W.L.Wendy Hsiao^1,4^*

1. State Key Laboratory of Quality Research in Chinese Medicine, Macau University of Science and Technology, Macao, China

2. Clinical Research Center, Shantou Central Hospital, Shantou, China

3. Zhuhai MUST Science and Technology Research Institute, Zhuhai, China

4. Affiliated Foshan Maternity and Child Healthcare Hospital, Southern Medical University, Foshan, Guangdong, China

# Equally contributed to this work

* Corresponding to W.L Wendy Hsiao at: State Key Laboratory of Quality Research in Chinese Medicine, Macau University of Science and Technology, Avenida Wai Long, Taipa, Macau SAR. E-mail: [bowhsiao@gmail.com](mailto:bowhsiao@gmail.com)

This file contains Table S1-4 and Figure S1.

**Table S1. The primer sequences of qPCR assays**

| **Target gene** | **Primer sequences (5’ to 3’)** | |
| --- | --- | --- |
|  | **Forward** | **Reverse** |
| *Bifidobacterium animalis* | GCGTGCTTAACACATGCAAGTC | CACCCGTTTCCAGGAGCTATT |
| E-Cadherin | GGACTACGATTATCTGAACG | AACACACACACTATCCAGC |
| FOXP3 | CCCATCCCCAGGAGTCTTG | ACCATGACTAGGGGCACTGTA |
| ICAM-1 | CGCTGTGCTTTGAGAACTGT | AGGTCCTTGCCTACTTGCTG |
| IL-1β | GCTGAAAGCTCTCCACCTCA | GGCCACAGGTATTTTGTCGT |
| IL-4 | GGTCTCAACCCCCAGCTAGT | GCCGATGATCTCTCTCAAGTGAT |
| IL-6 | CTTCCATCCAGTTGCCTTCTTG | AATTAAGCCTCCGACTTGTGAAG |
| IL-10 | GCTCTTACTGACTGGCATGAG | CGCAGCTCTAGGAGCATGTG |
| IL-12 | ACTCTGCGCCAGAAACCTC | CACCCTGTTGATGGTCACGAC |
| IL-13 | GGATATTGCATGGCCTCTGTAAC | AACAGTTGCTTTGTGTAGCTGA |
| IL-18 | CCTGACATCTTCTGCAACCT | TTCCGTTACTGCGGTTGT |
| iNOS | GTTCTCAGCCCAACAATACAAGA | GTGGACGGGTCGATGTCAC |
| *Lactobacillus johnsonii* | AGCAGTAGGGAATCTTCCA | CACCGCTACACATGGAG |
| N-Cadherin | GCGAGCCAGCAGATTTCAAG | TTGCCTGCTCTGCAGTGAGA |
| Occludin | ATGGCAAGCGATCATACCC | TTCCTGCTTTCCCCTTCG |
| TNF-α | CAAATGGCCTCCCTCTCAT | CTCCTCCACTTGGTGGTTTG |
| VCAM-1 | GAACCCAAACAGAGGCAGAG | GGTATCCCATCACTTGAGCAG |
| ZO-1 | AATGAATGATGGTTGGTATGG | TGACAGGTAGGACAGACG |

**Table S2: The formulation of the basic bacterial growth medium**

| **Ingredients** | **Grams per liter** |
| --- | --- |
| Acid Hydrolysate of Casein | 0.24 |
| Agar | 13 |
| Beef heart | 38.4 |
| Calf brain | 30.76 |
| Dextrose | 0.87 |
| Dipotassium Phosphate | 1.42 |
| Disodium phosphate | 0.43 |
| Magnesium Sulfate Heptahydrate | 0.024 |
| Pancreatic digest of casein | 1.7 |
| Piapiac digest of soybean meal | 0.3 |
| Protease peptone | 2.16 |
| Sodium chloride | 1.58 |
| Sodium Pyruvate | 0.14 |
| Soluble Starch | 0.24 |
| Yeast Extract | 0.24 |

**Table S3. The relative abundance of potential pathogens**

| **Species** | **Ctrl** | **PCP** | **5-FU** | **PCP+5-FU** |
| --- | --- | --- | --- | --- |
| *Akkermansia muciniphila* | 0.28±0.23 | 1.67±1.44 | 7.51±4.23 | 0.05±0.03 |
| *Alistipes finegoldii* | 1.53±0.68 | 0.56±0.08 | 3.17±0.26 | 2.73±1.1 |
| *Alistipes indistinctus* | 0.01±0.01 | 0 | 0.02±0.01 | 0.02±0.02 |
| *Alistipes massiliensis* | 4.81±2.13 | 2.42±0.98 | 3.53±3.43 | 1.31±0.37 |
| *Alistipes putredinis* | 0.05±0.02 | 0.05±0.02 | 0.13±0.03 | 0.06±0.02 |
| *Alistipes senegalensis* | 0.01±0.01 | 0.01±0 | 0.01±0 | 0.04±0.01 |
| *Alistipes shahii* | 0.02±0.03 | 0.01±0.01 | 0.03±0.01 | 0.03±0.02 |
| *Alistipes sp.* | 0.07±0.05 | 0.05±0.02 | 0.04±0.02 | 0.05±0.02 |
| *Alistipes spp.* | 0.32±0.27 | 0.17±0.09 | 0.2±0.13 | 0.18±0.09 |
| *Allobaculum sp* | 0.1±0.06 | 0.5±0.18 | 0.49±0.1 | 0.86±0.62 |
| *Allobaculum stercoricanis* | 0.07±0.05 | 0.86±0.58 | 0.13±0.1 | 0.49±0.24 |
| *Anaeroplasma spp.* | 1.15±0.69 | 0.65±0.37 | 1.73±0.26 | 1.55±0.46 |
| *Bacteroides vulgatus* | 0.91±0.72 | 0.03±0.01 | 1.49±0.99 | 1.94±0.36 |
| *Bilophila wadsworthia* | 0.02±0.02 | 0.02±0.01 | 0.03±0 | 0.03±0.01 |
| *Candidatus alistipes marseilloanorexicus* | 0.03±0.01 | 0.03±0.02 | 0.07±0.03 | 0.04±0.01 |
| *Citrobacter spp.* | 0.15±0.06 | 0.14±0.01 | 0.25±0.09 | 0.07±0.03 |
| *Clostridium sp.* | 3.57±0.08 | 2.68±0.34 | 4.4±1.47 | 3.4±0.49 |
| *Clostridium spp.* | 1.49±0.11 | 1.35±0.16 | 2.06±0.42 | 1.96±0.15 |
| *Clostridium sulfatireducens* | 0.98±0.11 | 0.99±0.21 | 1.15±0.47 | 0.99±0.14 |
| *Desulfovibrio desulfuricans* | 0.02±0.01 | 0.03±0.01 | 0.03±0.02 | 0.02±0.01 |
| *Desulfovibrio spp.* | 3.16±0.36 | 3.5±0.73 | 4.53±2.03 | 4.4±0.93 |
| *Dorea formicigenerans* | 0.02±0.01 | 0.03±0.02 | 0 | 0 |
| *Filifactor spp.* | 0.02±0.01 | 0.01±0 | 0.03±0.02 | 0.02±0 |
| *Filifactor villosus* | 0 | 0 | 0.01±0.01 | 0.01±0 |
| *Helicobacter apodemus* | 4.7±1.04 | 4.18±0.43 | 2.51±0.37 | 3.5±0.64 |
| *Helicobacter ganmani* | 1.72±0.72 | 1.77±1.61 | 0.85±0.75 | 1.4±0.6 |
| *Helicobacter hepaticus* | 0.16±0.09 | 0.14±0.05 | 0.05±0.01 | 0.21±0.07 |
| *Helicobacter typhlonius* | 3.87±3.23 | 3.24±1.93 | 2.99±0.63 | 3.35±0.58 |
| *Lachnoclostridium clostridium aldenense* | 0.31±0.07 | 0.47±0.08 | 0.05±0.06 | 0.19±0.11 |
| *Lachnoclostridium clostridium bolteae* | 0.02±0.02 | 0.01±0.01 | 0.01±0.01 | 0.01±0.02 |
| *Lachnoclostridium clostridium hathewayi* | 0.02±0.01 | 0.05±0.07 | 0.03±0.02 | 0.07±0.05 |
| *Mucispirillum schaedleri* | 0.37±0.42 | 0.06±0.02 | 0.87±0.64 | 0.42±0.16 |
| *Mycoplasma sualvi* | 0.04±0.02 | 0.04±0.02 | 0.05±0.05 | 0.03±0.01 |
| *Parabacteroides distasonis* | 1.68±0.2 | 1.14±0.27 | 0.93±0.35 | 0.75±0.19 |
| *Parabacteroides goldsteinii* | 0.46±0.42 | 0.14±0.06 | 1.13±0.05 | 0.44±0.1 |
| *Shigella sonnei* | 0.01±0.01 | 0.01±0 | 0.33±0.25 | 0.25±0.18 |
| *Spirochaeta spp.* | 0.02±0.01 | 0.03±0.01 | 0.02±0.01 | 0.02±0.01 |

**Table S4. The relative abundance of beneficial bacteria**

| **Species** | **Ctrl** | **PCP** | **5-FU** | **PCP+5-FU** |
| --- | --- | --- | --- | --- |
| *Acetivibrio spp.* | 0.01±0.01 | 0.02±0.01 | 0.01±0.01 | 0.01±0 |
| *Anaerostipes butyraticus* | 0.02±0.02 | 0.05±0.03 | 0 | 0.03±0.02 |
| *Anaerostipes caccae* | 0.05±0.05 | 0.05±0.06 | 0 | 0.28±0.21 |
| *Bacteroides acidifaciens* | 13.12±3.07 | 15.51±2.03 | 5.03±0.83 | 6.78±1.77 |
| *Bacteroides dorei* | 0.01±0.02 | 0 | 0.02±0.02 | 0.01±0.01 |
| *Bacteroides oleiciplenus* | 0.02±0.01 | 0.01±0 | 0.5±0.29 | 0.5±0.2 |
| *Bacteroides sartorii* | 0.78±1.11 | 0.15±0.08 | 2.63±2.29 | 3.46±1.52 |
| *Bacteroides sp.* | 0.14±0.18 | 0.12±0.13 | 2.38±2.73 | 0.78±0.42 |
| *Bacteroides spp.* | 0.08±0.03 | 0.07±0.02 | 0.1±0.03 | 0.1±0.02 |
| *Bacteroides thetaiotaomicron* | 0±0.01 | 0 | 0.02±0.01 | 0.04±0.01 |
| *Bacteroides uniformis* | 0.49±0.21 | 0.31±0.25 | 0.44±0.68 | 0.41±0.26 |
| *Bacteroides xylanisolvens* | 0.1±0.08 | 0.05±0.02 | 0.13±0.03 | 0.17±0.1 |
| *Bacteroides xylanolyticus* | 0.33±0.21 | 0.52±0.34 | 0.04±0.03 | 0.5±0.27 |
| *Barnesiella intestinihominis* | 0.24±0.09 | 0.35±0.11 | 0.25±0.19 | 0.31±0.03 |
| *Barnesiella spp.* | 9.66±0.92 | 13.55±2.1 | 8.41±1.03 | 13.28±2.25 |
| *Bifidobacterium choerinum* | 0.06±0.06 | 0.19±0.12 | 0.07±0.06 | 0.18±0.09 |
| *Blautia luti* | 0.05±0.09 | 0.09±0.05 | 0 | 0.04±0.01 |
| *Blautia producta* | 0.35±0.3 | 0.37±0.06 | 0.08±0.02 | 0.36±0.23 |
| *Blautia spp.* | 0.02±0.02 | 0.06±0.04 | 0.02±0.01 | 0.05±0.04 |
| *Butyricicoccus pullicaecorum* | 0.05±0.01 | 0.05±0.01 | 0.03±0.01 | 0.06±0.03 |
| *Butyrivibrio crossotus* | 0.03±0 | 0.01±0.01 | 0.03±0.02 | 0.02±0.02 |
| *Clostridium fusiformis* | 0.07±0.04 | 0.03±0.01 | 0.18±0.16 | 0.08±0.05 |
| *Eisenbergiella tayi* | 0.01±0.01 | 0.01±0 | 0 | 0±0.01 |
| *Eubacterium cellulosolvens* | 0 | 0.01±0.01 | 0.01±0.01 | 0 |
| *Eubacterium coprostanoligenes* | 0.05±0.03 | 0.18±0.11 | 0.07±0.02 | 0.16±0.12 |
| *Eubacterium desmolans* | 0.04±0.01 | 0.04±0.01 | 0.02±0 | 0.04±0.01 |
| *Eubacterium oxidoreducens* | 0 | 0 | 0.01±0 | 0.01±0.01 |
| *Eubacterium plexicaudatum* | 0.63±0.42 | 0.7±0.34 | 0.03±0.03 | 0.19±0.11 |
| *Eubacterium rectale* | 0.14±0.06 | 0.28±0.15 | 0.07±0.04 | 0.18±0.02 |
| *Eubacterium ruminantium* | 0.03±0.03 | 0.18±0.17 | 0 | 0 |
| *Eubacterium sp.* | 0.04±0.01 | 0.03±0.01 | 0.02±0.02 | 0.04±0.02 |
| *Eubacterium spp.* | 1.09±0.22 | 2.61±0.32 | 1.55±0.67 | 2.31±0.81 |
| *Eubacterium uniforme* | 0.03±0.02 | 0.03±0.01 | 0.04±0.02 | 0.02±0.01 |
| *Lachnoclostridium clostridium hylemonae* | 0.23±0.14 | 0.15±0.15 | 0.05±0.05 | 0.31±0.11 |
| *Lachnoclostridium clostridium indolis* | 0.58±0.36 | 0.25±0.19 | 0.3±0.19 | 0.54±0.08 |
| *Lachnoclostridium clostridium jejuense* | 0.02±0.01 | 0.01±0.01 | 0.04±0.05 | 0.02±0.01 |
| *Lachnoclostridium clostridium lavalense* | 0.35±0.31 | 0.39±0.05 | 0.46±0.41 | 0.42±0.18 |
| *Lachnoclostridium clostridium polysaccharolyticum* | 0.08±0.02 | 0.06±0.03 | 0.01±0 | 0.04±0.02 |
| *Lachnoclostridium clostridium saccharolyticum* | 1.22±0.57 | 1.72±1.12 | 0.43±0.3 | 0.6±0.12 |
| *Lachnoclostridium clostridium xylanolyticum* | 0.57±0.21 | 0.57±0.31 | 0.17±0.05 | 0.24±0.08 |
| *Lactobacillus johnsonii* | 0.06±0.07 | 0.04±0.03 | 0.02±0.01 | 0.09±0.04 |
| *Lactobacillus reuteri* | 0.02±0.03 | 0.02±0.01 | 0.02±0.01 | 0.03±0.02 |
| *Lactobacillus spp.* | 0.03±0.04 | 0 | 0 | 0 |
| *Lactococcus lactis* | 0.26±0.13 | 0.3±0.2 | 0.45±0.24 | 0.89±0.38 |
| *Prevotella sp.* | 2.41±0.48 | 2.23±0.86 | 1.53±0.71 | 1.94±0.95 |
| *Prevotella spp.* | 3.91±1.15 | 3.15±0.75 | 2.61±0.69 | 3.22±0.63 |
| *Pseudobutyrivibrio spp.* | 0.23±0.21 | 0.03±0.02 | 0±0 | 0.02±0.01 |
| *Roseburia faecis* | 0.1±0.03 | 0.06±0.02 | 0.02±0.01 | 0.08±0.04 |
| *Roseburia intestinalis* | 0.12±0.03 | 0.08±0.05 | 0.6±0.63 | 0.01±0.01 |
| *Roseburia sp.* | 0.01±0.01 | 0.08±0.07 | 0.1±0.09 | 0.12±0.1 |
| *Roseburia spp.* | 0.01±0 | 0.01±0 | 0.01±0.01 | 0.01±0 |
| *Ruminiclostridium clostridium leptum* | 0.09±0.05 | 0.03±0.01 | 0.14±0.05 | 0.1±0.12 |
| *Ruminiclostridium clostridium methylpentosum* | 0.06±0.04 | 0.03±0.02 | 0.05±0.04 | 0.02±0.01 |
| *Ruminiclostridium clostridium thermocellum* | 0.01±0 | 0 | 0.01±0 | 0 |
| *Ruminiclostridium eubacterium siraeum* | 0.06±0.07 | 0.27±0.23 | 0.19±0.07 | 0.29±0.23 |
| *Ruminococcus bromii* | 0.07±0.05 | 0.04±0.03 | 0.07±0.01 | 0.03±0.02 |
| *Ruminococcus flavefaciens* | 0.08±0.03 | 0.11±0.02 | 0.32±0.16 | 0.14±0.06 |
| *Ruminococcus sp.* | 0.16±0.06 | 0.32±0.12 | 0.08±0.03 | 0.09±0.02 |
| *Ruminococcus spp.* | 0.03±0.02 | 0.05±0.07 | 0.01±0.01 | 0 |
| *Tyzzerella clostridium lactatifermentans* | 0.02±0.01 | 0.01±0.01 | 0.04±0.02 | 0.06±0.04 |

**Figure S1:**


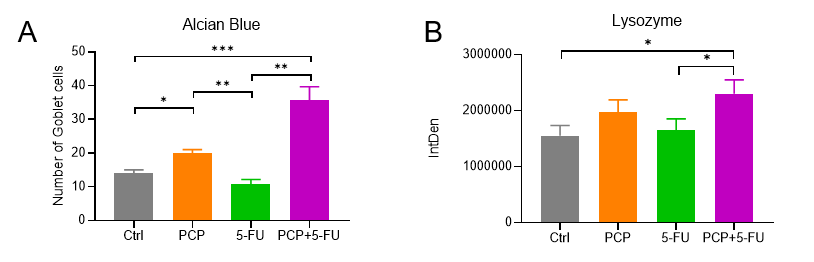


**Figure S1:** The number of Goblet cells and the quantitation of Paneth cells. The quantitation of Paneth cells was performed using Image J software, 374 * 264 pixel of positively stained areas were selected in each group for analysis. Data are presented as the mean ± SD, n=3. *p <0.05, ** p <0.01, *** p <0.001.
